# Pathogenic bacteria distributions and drug resistance analysis in 96 cases of neonatal sepsis

**DOI:** 10.1186/s12887-017-0789-9

**Published:** 2017-02-01

**Authors:** Huaifu Dong, Huiping Cao, Haiyan Zheng

**Affiliations:** 1 Department of Pediatrics, the First Affiliated Hospital of Bengbu Medical College, Bengbu, 233004 Anhui China; 2 Department of Pediatrics, the Third People’s Hospital of Bengbu, Bengbu, 233000 Anhui China; 3 Department of Nursing, the First Affiliated Hospital of Bengbu Medical College, Bengbu, 233004 Anhui China

**Keywords:** Neonatal sepsis, Pathogen, Drug resistance

## Abstract

**Background:**

This study aimed to summarize common pathogens and associated drug resistance in neonatal sepsis (NS).

**Methods:**

Blood culture and drug sensitivity results from 96 NS cases treated from January 2010 to August 2014 were retrospectively analyzed.

**Results:**

A total of 97 pathogenic bacteria were detected from these 96 NS cases; Gram-positive cocci accounted for 76.3% of the cases, among which 70.1% involved coagulase-negative staphylococcus (CONS), whereas Gram-negative bacilli and fungi accounted for 19.6% and fungi 4.1% of cases, respectively. Gram-positive cocci exhibited a higher penicillin resistance rate and full vancomycin sensitivity, whereas Gram-negative bacilli exhibited a higher cephalosporin resistance rate, low meropenem resistance rate (6.7%), and no resistance to amikacin.

**Conclusions:**

The main causative pathogens of NS in our hospital were Gram-positive cocci, among which coagulase-negative Staphylococcus spp such as S. epidermidis and S. haemolyticus were the main conditional pathogens; among Gram-negative pathogens, Klebsiella pneumoniae were most frequently isolated and showed widespread resistance to penicillins and cephalosporins.

## Background

Neonatal sepsis (NS) is an inflammation-induced systemic inflammatory response syndrome and an important cause of neonatal deaths [1, 2]; this condition comprises systemic poisoning symptoms caused by a large number of toxins produced by bacteria upon entry into the bloodstream, growth, and reproduction. As the neonatal blood–brain barrier is not completely developed, purulent meningitis is easily contracted and represents a great threat to the life and health of children, particularly preterm children [3]. NS is a serious infectious disease in the neonatal period and is mainly associated with immature neonatal immune functions. Early-onset sepsis (EONS) occurs within 3 days after birth; most pathogens derive from the mother's parturient canal and gastrointestinal tract, Gram-negative bacteria are the most frequently detected pathogens, and symptoms are normally severe. In contrast, late-onset sepsis (LONS) occurs 3 days after birth, with *Staphylococcus* spp. as the most frequently observed pathogens [4].

According to “Practical neonatology” by Shao, NS refers to bacterial and fungal infections, whereas Wang, in the National Teaching Material of Colleges and Universities, defines NS as involving bacterial, fungal, or protozoan pathogens. The present study has accepted the former concept. Despite the prophylactic application of antibiotics, NS currently remains an important cause of neonatal morbidity and mortality both in China and abroad [4].

Because NS has no specific clinical manifestations and blood culture analysis requires a long period of time, the positive blood culture rates among newborns in some developing countries were reported to be as high as 40–50%, whereas in China, Zhang reported a positive rate of 10.6%, and Zhang reported a rate of 12.03% [5, 6]. Although peripheral blood analysis can facilitate a diagnosis of sepsis [7, 8], the sensitivity and specificity are low, thus increasing the difficulty associated with clinical diagnosis and treatment; furthermore, NS is likely to be complicated by purulent meningitis, for which the prognosis was poor [9]. All of the above-mentioned factors require clinicians to provide a correct diagnosis and treatment for affected children as soon as possible to avoid unnecessary disability and death, and therefore an understanding of the pathogens and antibiotic resistance associated with the cases of NS admitted at our hospital and in nearby regions would be necessary. This study selected clinical data from 96 NS newborns with positive blood culture results who were treated in our department from January 2010 to August 2014 for a retrospective analysis.

## Methods

### Study information

Clinical data from a total of 96 NS cases (Neonates, 0–28 days) admitted to the neonatal intensive care unit (NICU) of Bengbu Third People’s Hospital from January 1, 2010 to August 31, 2014 because of positive blood culture results were collected. The risk factors for sepsis were trachea cannula, intravenously administered nutrition, and low response. Hematological parameters, pathogen features, major bacteria’s resistant organism detection rate, and antimicrobial resistance were reviewed from patients’ medical records. Invasive intervention such as endotracheal intubation and central venous catheter insertion were used in the neonates hospitalized.

### Diagnostic criteria

A diagnosis was made if patients exhibited clinical manifestations and met any one of the following criteria: (1) positive culture of pathogens from a blood culture or sterile body cavity culture; or (2) in cases with a positive blood culture result for an opportunistic pathogen (such as *coagulase negative staphylococcus aureus*, *klebsiella pneumoniae* etc.), the same type of bacteria must have been cultivated from another sample collected from the bloodstream, a sterile body cavity, or catheter tip.

### Clinical diagnosis

A clinical diagnosis was made if a patient exhibited clinical manifestations and had any of the following criteria: (1) non-specific examination with ≥2 items, including a white blood cell count (WBC) reduction to <5 × 10^9^/L; increase in WBC for ≤3 days to >25 × 10^9^/L, an increase in WBC for >3 days to >20 × 10^9^/L; immature/total neutrophil ratio (I/T) ≥0.16, ≥8 μg/ml, platelet count (PLT) ≤100 × 10^9^/L, and erythrocyte sedimentation rate ≥15 mm/h); or (2) positive pathogen antigen or DNA test results in a blood sample.

### Clinical data

Data from a total of 96 NS newborns with positive blood culture results who met the diagnostic criteria for sepsis were obtained from the medical record room and included each patient’s name, gender, age at admission, clinical manifestations of onset, laboratory examination findings, blood culture results, and drug sensitivity analysis results.

### Blood culture

For blood culture, the BACT/ALERT 3D 240 automatic blood culture system and associated flasks (BioMerieux Co., Lyon, France) were used; detected bacteria were also identified with an ATB Expression bacterial identification/sensitivity analyzer (BioMerieux Co.). WBC and PLT were determined on a Mindray BC-6800 automatic blood cell analyzer (Shenzhen Mindray Medical International Co., Ltd., Shenzhen, China). Serum CRP level was measured on a VITROS 250 automatic dry chemical biochemical analyzer (Johnson & Johnson, Shanghai, China).

### Antibiotic sensitivity analysis

Overnight fresh pathogen culture (log-phase broth culture) was flooded onto solid medium plates with the identified strains, and excess culture was removed. The plates were left at 25 °C for 24 h under sterile conditions. The antibiotic discs were placed and the culture plates were incubated at 37 °C for 24–48 h. The plates were examined under a stereo microscope (Leitz) and zones of inhibition were measured in millimeters from the edge of the disc.

### Statistical analysis

SPSS21.0 software (SPSS, Inc., Chicago, IL, USA) was used for the statistical analysis. Quantitative data were expressed as percentages or rates, inter-group comparisons were conducted using the *χ* ^2^ test and Fisher's exact test with a significance level of α = 0.05, and a *P*-value <0.05 was considered statistically significant.

## Results

### General information

This study included a total of 96 NS cases and isolated a total of 97 pathogenic strains (two strains were cultured from one sample); the cases included 68 boys (70.8%) and 28 girls (29.2%), for a boy/girl ratio of 2.4:1. Among the cases, 22 involved preterm births (22.9%), 74 involved full-term births (77.1%), and no cases involved post-term birth. The youngest patient at admission was 0.5 h, whereas the oldest was 26 days. Nineteen cases involved low birth-weight children (19.8%), including 12 cases of very low birth-weight children (12.5%), whereas 77 cases involved normal birth-weight children (80.2%). Forty-one cases involved early-onset NS (57.3%), and 55 involved of late-onset NS (42.7%); additionally, 66 cases achieved a complete healing (68.8%), 30 symptom improvement, and no deaths were observed. Lumbar puncture and cerebrospinal fluid (CSF) examination were performed in 23 NS cases, and the CSF findings of 7 cases were abnormal. In the study, there were no statistically significant differences between WBC, lymphocyte count, neutrophil count, and CRP level between EONS and LONS (*P* > 0.05).

### Pathogens distribution

Among the 96 cases, 97 pathogenic strains were identified, including 74 strains of Gram-positive cocci, 19 strains of Gram-negative bacteria, and 4 strains of fungi, accounting for 76.3%, 19.6%, and 4.1% of cases, respectively. Among the Gram-positive bacteria, *S. epidermidis* accounted for 44.3% (43/97) of cases, *S. haemolyticus* accounted for 14.4% (14/97), *S. hominis* accounted for 8.2% (8/97), and *S. aureus* accounted for 2.1% (2/97), whereas other bacteria (including *Kocuria varians*, *Listeria monocytogenes*, *Enterococcus faecalis*, *S. equorum*, and *S. bovis*) occurred at low rates. Additionally, among the 97 pathogens, 68 strains of coagulase-negative *Staphylococcus* (CONS) were detected. Among Gram-negative bacteria, *Klebsiella pneumoniae* subspecies accounted for 10.3% of cases (10/97) and *Escherichia coli* accounted for 6.2% (6/97); additionally, 2 strains of *Enterobacter cloacae* were detected along with 1 strain of *Sphingosine monad* and 4 types of fungi (*Candida glabrata*, 2 strains; *C. albicans* and *C. tropicalis*, had 1 strain, each). The specific composition is presented in Table 1.

**Table 1:** Distributions and constitutions of bacterial strains detected in 97 NS cases

Bacteria	Name	Cases	Percentage
Gram-positive bacteria		74	76.3%
	*Kocuria varians*	1	1.0%
	*S. epidermidis*	43	44.3%
	*L. Monocytogenes*	1	1.0%
	*E. faecalis*	1	1.0%
	*S. aureus*	2	2.1%
	*S. equorum*	1	1.0%
	*S. simulans*	1	1.0%
	*S. bovis*	1	1.0%
	*S. hominis*	8	8.2%
	*S. haemolyticus*	14	14.4%
	*S. warneri*	1	1.0%
Gram-negative bacteria		19	19.6%
	*E. coli*	6	6.2%
	*Klebsiella pneumoniae subsp.*	10	10.3%
	*S. monad*	1	1.0%
	*E. cloacae*	2	2.1%
Fungi		4	4.1%
	*C. albicans*	1	1.0%
	*C. glabrata*	2	2.1%
	*C. tropicalis*	1	1.0%

### Drug resistance in pathogens

Among the 74 cases of Gram-positive cocci, 43 strains of *S. epidermidis* were detected, as well as 14 strains of *S. haemolyticus* and 17 other strains. The total resistance rates to the following drugs were high among Gram-positive cocci: penicillin, 93.2%; ampicillin, 90.0%; oxacillin, 84.7%; erythromycin, 81.1%; and SMZ, 63.0%; as well as ciprofloxacin, 50.0%; levofloxacin, 35.0%; teicoplanin, 22.2%; fusidic acid, 18.0%; and rifampicin, 6.8%. No vancomycin-resistant Gram-positive bacteria were detected. Among the 43 strains of *S. epidermis*, the resistance rates were as follows: rifampin, 7.0%; fusidic acid, 10.7%; and teicoplanin, 24.3%. All 14 strains (100%) of *S. haemolyticus* exhibited full resistance to penicillin, erythromycin, and ampicillin, whereas the rates of resistance to oxacillin, levofloxacin, gentamicin, ciproflaxin, teicoplanin, and fusidic acid were 92.9%, 81.8%, 85.7%, 66.7%, 33.3%, and 27.3%, respectively. The remaining 17 strains of Gram-positive cocci were completely resistant to erythromycin and exhibited relatively higher rates of resistance to penicillin, oxacillin, SMZ, and ampicillin of 82.4%, 80%, 62.5%, and 66.7%, respectively; details are shown in Table 2.

**Table 2:** Drug resistance of Gram-positive bacteria [% (cases)]

	S. epidermidis	S. haemolyticus	Other 17 strains	Sum
Penicillin	95.3 (41/43)	100.0 (14/14)	82.4 (14/17)	93.2 (69/74)
Oxacillin	83.7 (36/43)	92.9 (13/14)	80.0 (12/15)^a^	84.7 (61/72)
Erythromycin	67.4 (29/43)	100.0 (14/14)	100.0 (17/17)	81.1 (60/74)
Levofloxacin	28.6 (10/35)^a^	81.8 (9/11)^a^	14.3 (2/14)^a^	35.0 (21/60)
Vancomycin	0.0 (0/43)	0.0 (0/14)	0 (0/17)	0.0 (0/74)
Rifampicin	7.0 (3/43)	7.1 (1/14)	5.9 (1/17)	6.8 (5/74)
Teicoplanin	24.3 (9/37)^a^	33.3 (4/12)^a^	7.1 (1/14)^a^	22.2 (14/63)
Ampicillin	100.0 (5/5)	100.0 (2/2)^a^	66.7 (2/3)^a^	90.0 (9/10)
Gentamicin	37.2 (16/43)	85.7 (12/14)	56.2 (9/16)^a^	50.7 (37/73)
SMZ	60.5 (26/43)	57.1 (8/14)	62.5 (10/16)^a^	60.3 (44/73)
Fusidic acid	10.7 (3/28)^a^	27.3 (3/11)^a^	27.3 (3/11)^a^	18.0 (9/50)
Ciprofloxacin	44.4 (4/9)^a^	66.7 (2/3)^a^	50.0 (2/4)^a^	50.0 (8/16)

Note: 1. ^a^: partial strains were not tested sensitivity to this drug, the denominator inside parentheses was the actual bacterial strains tested sensitivity to this drug. 2. The drug-resistant strains in this study referred to those with drug resistance (R) and interim strain (I)

Among the 19 Gram-negative bacterial strains, 6 were identified as *E. coli*, 10 were *Klebsiella pneumoniae* subsp., and 3 were other Gram-negative bacteria (2 strains of *E. cloacae* and 1 strain of *S. monad*). The Gram-negative bacterial strains exhibited the following high drug resistance rates: amoxicillin, 93.3%; ticarcillin, 93.3%; piperacillin, 75.0%; cefoxitin, 73.3%; amoxicillin/clavulanate potassium, 73.3%; ticarcillin/clavulanate, 73.3%; piperacillin/tazobactam, 61.1%; cefuroxime, 75.0%; ceftazidime, 73.7%; cefepime, 73.7%; cefalotin, 66.7%; and cefotaxime, 68.8%. In addition, these strains exhibited rates of resistance to gentamicin tobramycin, imipenem, and meropenem of 42.1, 33.3, 21.1, and 6.7%, respectively, whereas no strains were resistant to amikacin. None of the 6 strains of *E. coli* were resistant to meropenem, imipenem, or amikacin, whereas relatively high resistance rates to amoxicillin and SMZ of 83.3% and 66.7%, respectively, were observed; in addition, 33.3% of strains were each resistant to cefoxitin, ceftazidime, and cefepime, whereas 16.7% were resistant to piperacillin/tazobactam. The 10 strains of *K. pneumoniae* subsp. exhibited full resistance (100%) to cefuroxime, cefoxitin, ceftazidime, cefepime, amoxicillin/clavulanate, cefotaxime, amoxicillin, and piperacillin, a 70.0% cephalothin resistance rate, and no resistance to meropenem, amikacin, or netilmicin. The other 3 Gram-negative bacterial strains (2 strains of *E. cloacae* and 1 strain of *S. monad*) exhibited total resistance to amoxicillin, piperacillin/tazobactam, cefoxitin, amoxicillin/clavulanate, cephalothin, and tobramycin, but full sensitivity to amikacin (Table 3).

**Table 3:** Drug resistance of gram-negative bacilli [% (cases)]

	E. coli	Klebsiella pneumoniae subsp.	Others	Sum
Gentamicin	33.3 (2/6)	40.0 (4/10)	66.7 (2/3)	42.1 (8/19)
SMZ	66.7 (4/6)	50.0 (5/10)	33.3 (1/3)	52.6 (10/19)
Amoxicillin	83.3 (5/6)	100.0 (7/7)	100.0 (2/2)	93.3 (14/15)
Piperacillin	50.0 (3/6)	100.0 (7/7)^a^	66.7 (2/3)	75.0 (12/16)
Piperacillin/Tazobactam	16.7 (1/6)	80.0 (8/10)	100.0 (2/2)^a^	61.1 (11/18)
Cefoxitin	33.3 (2/6)	100.0 (7/7)^a^	100.0 (2/2)^a^	73.3 (11/15)
Ceftazidime	33.3 (2/6)	100.0 (10/10)	66.7 (2/3)	73.7 (14/19)
Cefepime	33.3 (2/6)	100.0 (10/10)	66.7 (2/3)	73.7 (14/19)
Cefuroxime	50.0 (3/6)	100.0 (7/7)	66.7 (2/3)	75.0 (12/16)
Meropenem	0.0 (0/6)	0.0 (0/7)^a^	50.0 (1/2)^a^	6.7 (1/15)
Imipenem	0.0 (0/6)	30.0 (3/10)	33.3 (1/3)	21.1 (4/19)
Amikacin	0.0 (0/6)	0.0 (0/10)	0.0 (0/3)	0.0 (0/19)
Amoxicillin/clavulanate	33.3 (2/6)	100.0 (7/7)^a^	100.0 (2/2)^a^	73.3 (11/15)
Cefalotin	50.0 (3/6)	70.0 (7/10)	100.0 (2/2)^a^	66.7 (12/18)
Cefotaxime	33.3 (2/6)	100.0 (7/7)^a^	66.7 (2/3)	68.8 (11/16)
Tobramycin	50.0 (3/6)	10.0 (1/10)	100.0 (2/2)	33.3 (6/18)

Note: 1. ^a^: partial strains were not tested sensitivity to this drug, the denominator inside parentheses was the actual bacterial strains tested sensitivity to this drug. 2. The drug-resistant strains in this study referred to those with drug resistance (R) and interim strain (I)

Furthermore, 4 strains of fungi (2 strains of *C. glabrata*, 1 strain of French *C. albicans*, and 1 strain of *C. tropicalis*) were detected. All 4 strains were sensitive to 5-fluorocytosine and amphotericin B, whereas only 2 strains (1 strain each of *C. glabrata* and *C. tropicalis*) were resistant to fluconazole. Only 1 strain of *C. glabrata* was sensitive to itraconazole, whereas the remaining 3 strains were resistant; additionally, only the *C. tropicalis* strain was resistant to voriconazole, whereas the other 3 strains were sensitive.

## Discussion

Sepsis is a severe illness during the neonatal period and remains among the main causes of neonatal death. Children admitted to the NICU are normally in a serious condition or premature; the prevalence of sepsis among long-term hospitalized children may be as high as 30%, with a mortality rate as high as 50%, and survivors experience serious sequelae [8–10]. Given the concentrated spectra of pathogens, foreign countries often classify NS cases according to the age of onset as either EONS or LONS in order to inform the clinical selection of antibiotics and assess prognosis [11]. EONS mainly emphasizes that the infection occurred in utero or during birth and that the pathogens mainly originated in the maternal birth canal, whereas LONS normally occurs after birth. In China, there is current disagreement about the distinction between EONS and LONS, and this study accordingly did not distinguish cases using this classification. The main pathogens of NS reported among cases in China are Gram-positive bacteria, particularly *Staphylococcus* spp. [12]. Certain evidence has proven an increasing trend of *S. epidermidis* infection [13]. Among the 97 strains of bacteria, 68 strains of CONS were detected, accounting for 70.1% of the cases. Among Gram-negative bacteria, *K. pneumoniae* subsp. accounted for the highest proportion, 10.3% (10/97), followed by *E. coli* (6.2%, 6/97) and *E. cloacae*, whereas the overall detection rate was low and basically consistent with that reported by Tang, suggesting that the NS-causative pathogens detected in our department remained mainly Gram-positive cocci, especially coagulase-negative *S. epidermidis* and *S. haemolyticus*, followed by *K. pneumoniae* subsp., whereas the proportions of coagulase-positive *S. aureus* and *Streptococcus* were relatively lower; in contrast, *S. haemolyticus* B is the main pathogen in foreign countries [14], which might be related to geographical differences.

Currently, although the extensive application of antibiotics could quickly control infections or prevent NS to a limited extent and scope, it could also easily induce highly drug-resistant strains [13]. Therefore, when antimicrobial drugs are clinically applied, the principles of antibiotic application and issues related to antibiotic resistance should be strictly controlled, and drugs should be selected according to individual sensitivity to reduce bacterial resistance to antimicrobial drugs and improve drug efficacies. The supplementary issue of the Performance Standards for Antimicrobial Testing, 23rd edition mentioned that in addition to cephalosporins, which exhibit novel anti-MRSA activities, oxacillin-resistant *Staphylococcus* spp. also exhibited resistance to all β-lactam enzyme-type drugs [15]. The data in the present study revealed that 74 strains of Gram-positive cocci exhibited higher resistance rates to penicillin, ampicillin, oxacillin, and erythromycin while remaining fully sensitive to vancomycin. Common CONS such as *S. epidermidis* and *S. haemolyticus* exhibited penicillin and erythromycin resistance rates as high as 100%. Nineteen strains of Gram-negative bacilli exhibited amoxicillin resistance rates as high as 93.3% and resistance rates higher than 60% to commonly used antibiotics such as piperacillin, cefuroxime, ceftazidime, cefepime, and amoxicillin/clavulanate; in contrast, lower resistance to imipenem and meropenem was observed, and amikacin was the most efficacious drug. The most common strain, *Klebsiella pneumoniae*, was completely resistant to commonly used drugs such as penicillin and cephalosporins, exhibited the highest sensitivity rates to meropenem and amikacin, indicating that previously used penicillins, erythromycin, and cephalosporins could not be used as preferred medications for NS. Vancomycin was the most effective antibiotic against neonatal Gram-positive bacterial infections; although amikacin is completely effective against (the isolated) Gram-negative bacteria, it can induce severe ear and kidney toxicities and therefore has been rarely used for neonatal infection. The rates of resistance to carbapenems such as imipenem and meropenem were low and as these drugs rarely caused adverse reactions, they could be used as a first-line treatment for the prevention or treatment of neonatal Gram-negative bacillus infections. In recent years, given the widespread application of imipenem, vancomycin, and other antibiotics, as well as the continuous development of invasive procedures such as endotracheal intubation and central venous catheter insertion, reports about multi-resistant strains, such as those exhibiting anti-carbapenem and anti-vancomycin characteristics, have continually increased in China and abroad [16]. Although the resistance rates to imipenem, meropenem, and other carbapenems in this study were low, they could not be ignored. Therefore, clinicians should strictly control the conditions under which antibiotics are applied, minimize the use of invasive procedures, and replace antibiotics as necessary and in a timely manner according to the situation to reduce the incidence of drug resistance. Better medical decisions, particularly those for appropriate detection methods and initial antimicrobial therapies, can be made by understanding the different clinical features and causative pathogens in both EONS and LONS. The limitations of this study are the single medical center-restricted data and small sample size.

## Conclusion

The main causative pathogens of NS in our hospital were Gram-positive cocci, among which coagulase-negative Staphylococcus spp such as S. epidermidis and S. haemolyticus were the main conditional pathogens; among Gram-negative pathogens, Klebsiella pneumoniae were most frequently isolated and showed widespread resistance to penicillins and cephalosporins.

## Abbreviations

CONS: Coagulase-negative staphylococcus
EONS: Early-onset sepsis
LONS: Late-onset sepsis
NICU: Neonatal intensive care unit
NS: Neonatal sepsis
PLT: Platelet count
WBC: White blood cell count
